# Response of the Pre-Oriented Goal-Directed Attention to Usual and Unusual Distractors: A Preliminary Study

**DOI:** 10.18869/nirp.bcn.8.2.155

**Published:** 2017

**Authors:** Golnaz Baghdadi, Farzad Towhidkhah, Reza Rostami, Mohsen Raza

**Affiliations:** 1. Department of Biomedical Engineering, Amirkabir University of Technology, Tehran, Iran.; 2. Department of Psychology, School of Psychology and Education, University of Tehran, Tehran, Iran.; 3. Department of Neurology, Faculty of Medicine, Baqiyatallah University of Medical Sciences, Tehran, Iran.

**Keywords:** Unusual events, Orientation, Initial condition sensitivity, Distractors, Goal-directed attention

## Abstract

**Introduction:**

In this study, we investigated the distraction power of the unusual and usual images on the attention of 20 healthy primary school children.

**Methods:**

Our study was different from previous ones in that the participants were asked to fix the initial position of their attention on a predefined location after being presented with unusual images as distractors. The goals were presented in locations, which were far from the attraction basin of distractors. We expected that the pre-orienting of the attention to the position of targets would reduce the attractive effect of unusual images compared to the usual ones. The percentage of correct responses and the reaction time were measured as behavioral indicators of attention performance.

**Results:**

Results showed that using the goal-directed attention, subjects ignored both kinds of distractors nearly the same way.

**Conclusion:**

With regard to previous reports about more attraction towards the unusual images, it is suggested that the dynamics of the visual attention system be sensitive to the initial condition. That is, changing the initial position of the attention can lead to the decrement of the unusual images effects. However, several other possibilities such as a probable delay in processing unusual features could explain this observation, too.

## 1. Introduction

The impairment effects of distractors on attention were investigated intensively in previous studies. Distractors activate some neural processes that draw attraction to some unrelated objects (Schröger & Wolff, 1998). Goal-directed attention (i.e. attention to a predefined goal) helps ignore the distractions and minimizes their impairment effects. It was shown that the probability of success in this minimization depends on the features of the distraction. One of these features is the novelty. Previous studies have shown that novel (Folstein, Van Petten, & Rose, 2008; Parmentier, Ljungberg, Elsley, & Lindkvist, 2011; van Kesteren, Ruiter, Fernández, & Henson, 2012; Yang et al., 2009), surprising (Alho et al., 1998; Baldi, 2002; Dewald, Sinnett, & Doumas, 2013; Macedo & Cardoso, 2001), unpredictable (Berti & Schröger, 2003; Matthews, Scheier, Brunson, & Carducci, 1980), unfamiliar (Carver, Meltzoff, & Dawson, 2006; Nittono, Shibuya, & Hori, 2007), or unexpected events (Horstmann, 2005; Summerfield & Egner, 2009) can affect attention and memory performance in various ways. However, there are some special conditions that can eliminate the effect of novel distractions (e.g., presenting the distraction in an uninformative circumstance (Parmentier, Elsley, & Ljungberg, 2010; Wetzel, Widmann, & Schröger, 2012), forcing the subject’s attention back to the target (Parmentier, Elford, Escera, Andrés, & San Miguel, 2008), or increasing the working memory load (SanMiguel, Linden, & Escera, 2010).

It has been shown that valid or invalid orientation of the attention system has significant effect on its performance (Adler et al., 2014; Reis Lellis et al., 2013). If the subject’s attention is oriented to an invalid position, the attention performance drops dramatically (Abundis-Gutiérrez, Checa, Castellanos, & Rueda, 2014). In all attentional tasks, orienting cues determine the initial position of the focus of the attention before the onset of targets. Unlike previous studies on the effects of novel images, we fixed the initial focus of the subjects’ attention on the position of the appearance of targets. The usual and unusual distractors were presented in a place with the greatest possible distance from the targets. That is, if a separate basin of attraction was considered for targets and distractors, the participants would be asked to fix their attention focus on the basin of targets.

Therefore, the design of our experiment is different from visual search tasks in which the subject should find the place of a predefined goal between different randomly distributed distractors. The scenario of fix positioned goals and distractions, often happens for a student in a class. For example, students are always asked to focus on the board, listen to their teacher (a fixed position goal), and try to ignore every incoming disturbance from the class window (a fixed position distraction). Attention helps them ignore the irrelevant environment stimuli and reserve their cognitive processing resources to respond to their teacher’s commands. In our experimental design, we provided a similar situation.

The participants were asked to focus on a predefined place on a monitor screen (analogous to the board or teacher in the classroom). Different predefined target and nontarget shapes were presented in this place. The subjects should click a mouse when they see the predefined target (like responding to the teacher’s commands). Simultaneously, some usual or unusual images were presented as distractors on the opposite side of the predefined place (analogous to irrelevant distraction that comes from classroom’s window). This experiment is performed on some primary school children from both the genders.

In summary, the main goal of the current study is to find the effect of unusual distractors compared to usual ones when the initial position of the attention focus is far from the distractors. It can provide us more knowledge about the mechanism of the goal-directed visual attention in the presence of distraction. The subjects’ attentional performance was investigated by the changes of the reaction time and the correctness of responses.

In the next part, the explanation about the effect of initial position, the characteristics of our participants, and the experiment has been reported. Then, the recording parameters to study the subjects’ attentional performance are introduced followed by the obtained results of investigating the values of these parameters. Last part contains justification of the obtained result using prior findings and existent facts about our attention system.

### 1.1. Sensitivity to the Initial Condition (IC)

As it was mentioned, in our experiment, the subjects were asked to fix their attention focus on the place where the goals were presented. Therefore, we deliberately set the initial position of attention on the place where was near the goal and far from distractors. Hence, in the following subsection, we briefly explained the effect of Initial Condition (IC) in dynamic systems that can help us discuss the results of our experiment in the next sections.

**Figure 1.**
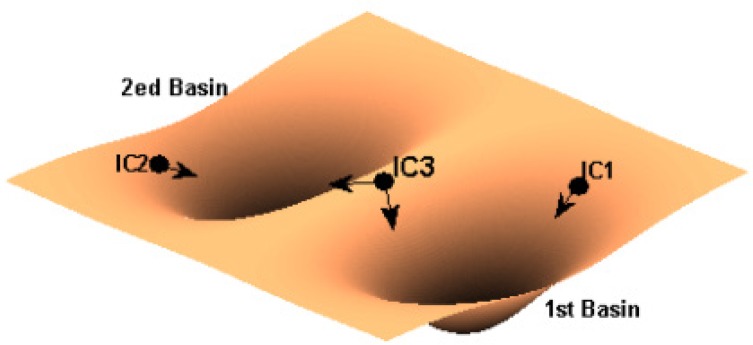
A system with two basins of attraction. The position of IC (i.e. IC1, IC2, and IC3) determines the final state of the system.

Sensitivity to IC is one of the characteristics of chaotic and complicated systems. This means that a tiny perturbation of the IC can lead to a different and unpredictable outcome. The IC effect will be more pronounced when there are several basins of attraction in the system. That is, based on the IC value, the system will be attracted by one of the basins in its final state. For instance, Figure 1 shows a system with two basins of attraction.

This figure indicates that when the IC is near the first basin (i.e. IC1), the system falls in this basin at its final state. However, when the IC is in the border of two basins (i.e. IC3), the system is attracted by one of them with higher level of attraction (e.g., steep slope). Therefore, changing the IC can force the system to switch from one outcome to another one. The sensitivity to IC has been observed in many natural and artificial systems (Brown, Chua, & Popp, 1992; Li & Gu, 2015; Mukougawa, Sakai, & Hirooka, 2005).

In next sections, we investigated the behavior of the attention control system in dealing with two kinds of distractions when the initial position of the subject’s attention is not near the attraction basin of distractions.

## 2. Methods

As it was mentioned, in our experiment, the subjects were asked to fix their attention focus on the place where the goals were presented. Therefore, we deliberately set the initial position of attention on the place where was near the goal and far from distractors. Hence, in the following subsection, we briefly explained the effect of Initial Condition (IC) in dynamic systems that can help us discuss the results of our experiment in the next sections.

Sensitivity to IC is one of the characteristics of chaotic and complicated systems. This means that a tiny perturbation of the IC can lead to a different and unpredictable outcome. The IC effect will be more pronounced when there are several basins of attraction in the system. That is, based on the IC value, the system will be attracted by one of the basins in its final state. For instance, Figure 1 shows a system with two basins of attraction.

This figure indicates that when the IC is near the first basin (i.e. IC1), the system falls in this basin at its final state. However, when the IC is in the border of two basins (i.e. IC3), the system is attracted by one of them with higher level of attraction (e.g., steep slope). Therefore, changing the IC can force the system to switch from one outcome to another one. The sensitivity to IC has been observed in many natural and artificial systems (Brown, Chua, & Popp, 1992; Li & Gu, 2015; Mukougawa, Sakai, & Hirooka, 2005).

In next sections, we investigated the behavior of the attention control system in dealing with two kinds of distractions when the initial position of the subject’s attention is not near the attraction basin of distractions.

### 2.1. The Experiment

In this experiment, we aimed to find the effect of distractions on the attention system when the IC of the system is out of the attraction basin of distractors. In our definition, the subject’s attention is on the distractor’s basin of attraction if a section of the distractor image is in the subject’s center of view. In this situation, this part of the distractor image attracts the attention and finds the opportunity to enter the subject’s working memory for further processing. In the experiment, distractors were presented in a position (i.e. the top left of the screen) with the greatest possible distance from the target position (i.e. the right bottom of the screen). We fixed the position of usual and unusual distractors in the experiments, and the participants were informed about the presentation position of the goals and asked to fix their gaze on this position. Therefore, the design of the experiment is so that forces the participants gaze to be far from the position (i.e. attraction basin) of distractors at the beginning of each trial (i.e. IC).

### 2.2. Practice and Testing Phase of the Task

**Figure 2.**
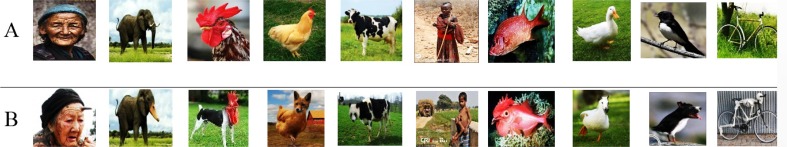
(A): Usual images, (B): Unusual images, these images were used as distractor in goal-directed attention task.

In order to answer the main question of the research, “what is the difference between goal-directed attention performance in ignoring usual and unusual distractors?” 20 images (10 usual and 10 unusual) were selected for presenting as distractors. The unusual images were the ones that no one had seen them in his or her life, for instance, an animal with the body of a hen and the head of a fox is unusual and unexpected. At the end of the test, the participated students were asked to say which images was unusual and unexpected. All of them claimed that the selected unusual images had been surprising. Corresponding to each unusual image, a usual image was selected. Figure 2 shows all 20 images that were gathered from different sites.

**Figure 3.**
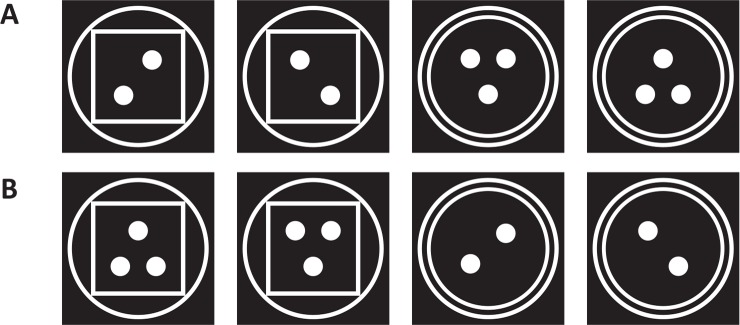
(A): Target items, (B): Nontarget items. The shapes of target and nontarget items were selected based on the Frankfurt adaptive concentration test (Goldhammer, Moosbrugger, & Krawietz, 2009).

These usual and unusual images were presented as distractors that could interfere with the proper response to the target items. The subject was requested to click on a button as soon as he/she observed the target item that was defined as “circles with three dots” and “squares with two dots” (Figure 3A). On the other hand, the subject should inhibit his or her response against nontarget items that were defined as “circles with two dots” or “squares with three dots” (Figure 3B). The target and nontarget images were selected based on the “Frankfurt Adaptive Concentration Test” (Goldhammer, Moosburgger, & Krawietz, 2009). This test has been introduced to investigate the subjects’ concentration capacity. Since this test demands high level of attention (i.e. high cognitive load), we choose it to increase the sensitivity to the distraction.

#### 2.2.1. Practice phase

**Figure 4.**
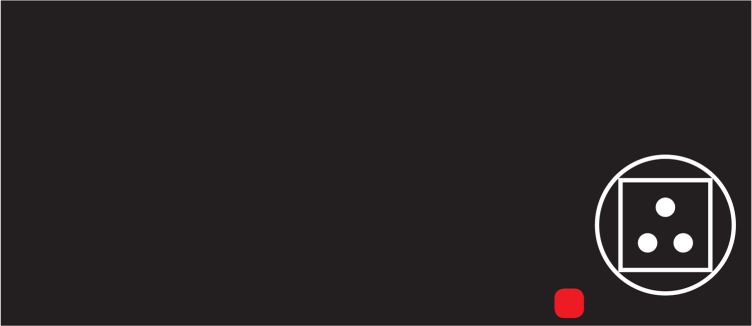
A sample trial of the practice phase of the task that contains a nontarget item on the right bottom of the screen. A button was considered near the item that the subject should click on it for responding to targets.

To ensure that the children can distinguish the target and nontarget items, a practice phase was considered before performing testing phase of the task. Practice phase consists of trials that one of the target or nontarget items was presented on the right bottom of them (Figure 4). The subject was requested to respond to the target items by left clicking on the red square, which is located near the presented item, with the mouse (Figure 4). No response was requested for nontarget items, and no distractor was presented in this phase.

An appropriate audio feedback was used to inform the subject from his or her wrong and correct response. This phase continued until the children ensured about his or her ability to detect the target and nontarget item. Therefore, we have ensured that the observed wrong answers in the testing phase were probability because of distraction not incomplete understanding of the problem.

#### 2.2.2. Testing phase

**Figure 5.**
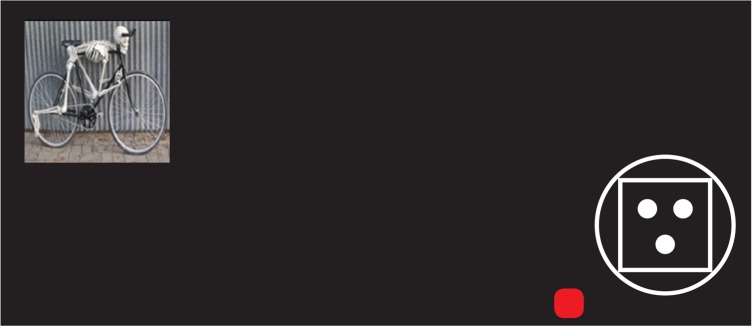
A sample trial of the task that contains a nontarget item on the right bottom of the screen, near the button that the subject should click on it for responding to the target items and one of the unusual distractor on the top left of the screen.

Testing phase of the task consists of 30 trials. In each trial, target or nontarget items were presented on the right bottom of the screen, near the button that the subject used for responding to target. In some trials, one of the usual or unusual images was shown on the top left of the distraction (Figure 5). The right bottom of the screen is a place where the right-handed subjects can look and concentrate on it with the least pressure on the eye muscles and the motor system. Therefore, we fixed the place of the target presentation on the right bottom of the screen. Hence, the chance of ignoring the target place, due to the eye muscles fatigue, decreases. Distractions have been also presented in a place with the greatest distance (i.e. top left) to the target point to increase the demand of saccade for detailed processing of the distraction and consequently, leads to the further increase of the reaction time.

In general, 5 trials contained a target item and one of the usual distractors; 5 trials contained a target item and one of the unusual distractors; 5 trials contained a nontarget item and one of the usual distractors; 5 trials contained a nontarget item and one of the unusual distractors. Five trials contained a target item with no distractors; and 5 trials contained a nontarget item with no distractor. Therefore, trials of the experiments included 6 conditions. The first two trials had no distractor. In each trial, the images were presented for 1500 ms. This is a period that contains only the working memory processes (Mate, Pires, Menéndez, & Estaún, 2009). The interval between images was set at 1500 ms to decrease the effect of two successive images on each other (Jiang, 2004).

We wanted the children to put their concentration on the right bottom of the screen to respond for target and nontarget items as fast and accurate as possible. Task duration, including clicking speed measurement, of the practice and testing phase, were approximately between 5 to 10 minutes. Table 1 shows the details of the duration of each phase in the task.

**Table 1.** The duration of each phase in the task.

**Phase**	**Duration**
Clicking speed measurement	1 Minute
Practice	Depending on the subject
Test	2 Minutes
Total	5 to 10 Minutes

The stimuli were presented on a 14.6′ LCD screen with the resolution of 1600×900 pixels and the refresh rate of 60 Hz. The distance of participants from the screen was about 70 cm. During the testing phase, a camera was placed under the monitor and tuned to record the face of the subject.

The subjects were requested to sit on the seat and do not change their head and body position in the test phase. The output of the camera was used to track the subjects’ head and eyes position and ensured us about the participants’ fixed and appropriate situation. Distractors were presented on the left-top of the screen by the dimension of 324×308 pixels. Target or nontarget items were shown on the right-bottom of the screen by the dimension of 269×252 pixels.

A program designed in Visual C# running under Microsoft windows 7 controlled the presentation and storage of different parameters.

### 2.3. Recorded parameters in the testing phase of the task

Three variables have been measured in this study: 1) the correct excitatory response: the number of clicking on the button in the presence of target items, 2) the correct inhibitory response: The number of not clicking on the button in the presence of nontarget items, and 3) the reaction time of correct answers was recorded during the testing phase of the task (the measurement of correct inhibitory responses’ reaction times is not possible). Reaction time has been defined as the duration between the onset of the target item and the time of pressing the button by the subject.

If the subjects’ attention shifted to distraction in the early moments of the interval (i.e. 1500 ms of image presentation), the reaction time increases to have a correct excitatory response. Therefore, the higher values of the reaction time in correct responses can be considered as a sign of the attraction by the distraction. The correct inhibitory or excitatory responses are also expected to be affected by the distraction, because the subjects are requested to respond as fast and accurate as possible. It was shown that people tend to have impulsive behavior (i.e. making more errors) in the presence of the distraction (Beaman, Hanczakowski, & Jones, 2014). That is, in the trade-off between the speed and the accuracy, they put more weight on the speed that consequently, leads to incorrect responses.

## 3. Results

Our research aimed to investigate the subjects’ attentional performance in ignoring usual and unusual distractors when the initial position of the attention focus was located far from the attraction basin of distractors. To find the answer of the research question, the obtained values of each criterion (the correct inhibitory and excitatory responses and the reaction time) were investigated separately for usual, unusual, and no distractor using Friedman test at the significance level of 0.05 (The distribution of data in different conditions was not normal).

### 3.1. Correct responses (Inhibitory and excitatory)

**Figure 6.**
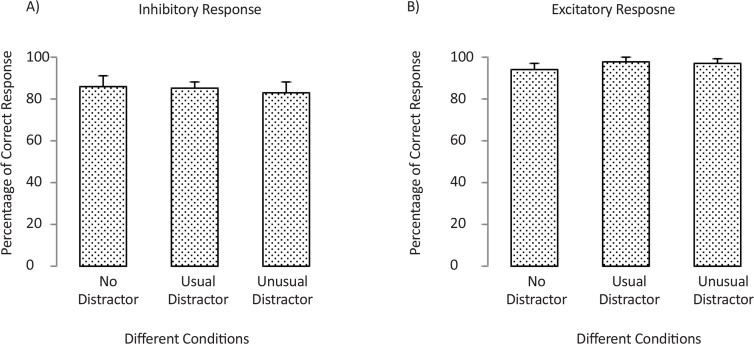
Percentage of correct inhibitory response to nontarget items (A) and correct excitatory response to targets (B) in trials with usual distractors, unusual distractors, and with no distractor. Error bars show the standard error of mean.

With regard to the effect of distraction type on the percentages of the correct responses, means and standard error of means (SEM) of the percentage of correct excitatory (E) and inhibitory (I) responses in trials with usual, unusual, and with no distractor are presented in Table 2. The bar graph of the data in each condition is plotted in Figure 6.

**Table 2.** Percentage of correct excitatory (E) and inhibitory (I) responses in trials with usual, unusual, and with no distractor.

	**No Distractor**		**Usual Distractor**		**Unusual Distractor**	

	**I**	**E**	**I**	**E**	**I**	**E**
Mean (SEM)	86(6.2)	94(4.6)	85(6.1)	98(1.9)	83(5.1)	97(2.3)

Non-parametric Friedman test was conducted to compare the effect of distraction type (usual, unusual, and with no distractors) on the percentage of inhibitory correct response. Results showed no significant main effect for the factor of distraction type (Chi-square=0.53, P=0.76). A similar analysis was repeated on the percentage of excitatory correct response. Results showed that the distraction type had no significant effect (Chi-square=2, P=0.36). Therefore, the correct response of participants in trials with usual, unusual, and no distractor was almost similar.

### 3.2. Reaction Time (RT)

**Figure 7.**
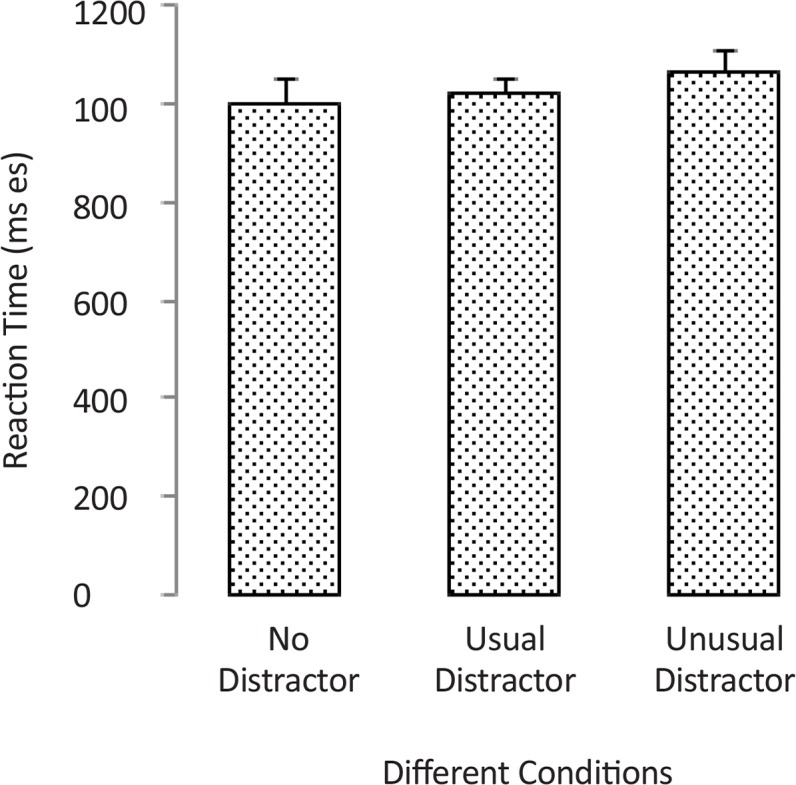
The reaction time of correct excitatory responses (clicking on the button in the presence of target items) in trials with usual, unusual, and no distractor.

With regard to the effect of the distraction type on reaction time (RT), Table 3 presents the abstract statistical information of reaction times (RTs) in different conditions. Figure 7 shows the bar graph of RT values. Using the non-parametric Friedman test, the effect of distraction type (usual, unusual, and with no distractors) on the RT values was investigated. Results showed no significant main effect for the factor of distraction type (Chi-square=2.8, P=0.24). Therefore, the measured values of RTs in trials with usual, unusual, and no distractor were almost similar. However, Table 3 shows that on average the value of RT in trials with unusual distractor is higher than others.

**Table 3.** The reaction time of correct excitatory responses (clicking on the button in the presence of target items) in trials with usual, unusual, and no distractor.

	**No Distractor**	**Usual Distractor**	**Unusual Distractor**
Mean (SEM) ms	998.9(49.6)	1021(41.3)	1062(52)

## 3.3. Power analysis

The sample sizes of the previous studies on the effect of novel or unusual images, which were introduced in the introduction section, were almost similar to our study. However, the statistical power analysis was done using the G*Power software (for more details refer to Faul, Erdfelder, Buchner, & Lang, 2009; Faul, Erdfelder, Lang, & Buchner, 2007) to check whether our non-significant outcomes were due to a weak statistical power.

The analysis showed for inhibitory correct response, excitatory correct response, and the reaction time the sample sizes of N=278787, 8025, and 6909 subjects would be needed, respectively to find statistical significance results with 80% power (1-β) at 0.05 level. Because of the need to very large sample sizes, the difference between the effect of the usual and unusual distractors on the goal-directed attention is extremely small.

### 3.4. Correlation analysis

To investigate the relationship between the variables (percentage of correct responses and the reaction time), the non-parametric Spearman correlation was calculated.

The results of the analysis showed that the correlation between variables was not significant in all conditions, unusual (r=−0.4, P=0.07), usual (r=−0.4, P=0.1), and no distractors (r=−0.3, P=0.18). However, the negative correlation between the variables supports the competition and the required trade-off between the speed and accuracy of the response.

## 4. Discussion

Based on different behavioral indicators, we investigated the performance of the visual goal-directed attention in the presence of usual and unusual distractors when the initial focus of attention was near the attraction basin of the goal. According to the effect of initial condition in complex systems, the reduction of the impairment effect of unusual images on attention performance was expected. Results confirmed our expectation and showed both usual and unusual distractors had the same effect on the performance of goal-directed attention.

That is, both kinds of distractors capture the same amount of attention, or the subject goal-directed attention responded almost similarly to both kinds of distractions. Our results are apparently inconsistent with the findings of previous studies that reported novel (unusual) events can interrupt ongoing mental process, change the orientation of attention, and make delay in motor response even in newborns (Folstein, Van Petten, & Rose, 2008; Mather, 2013). However, it should be noted that in those studies, novel stimuli were not presented as distractors, the presentation location of novel or unusual stimuli was not far from the focus of attention, and the subjects were not requested to fix their initial position of their attention on a place that had a considerable distance from the attraction basin of distractors. In other words, if our participants were asked to attend to usual or unusual images instead of focusing on the target’s location, attention to unusual images may be preferred due to their novel features.

In summary, comparing our results with the outcomes of the previous results showed that if the subject was asked to fix the initial position of his or her attention on a predefined target place, there would be no significant difference between the effect of usual and unusual distractors (First Scenario). However, if the subject is not oriented to the target position before the appearance of the disturbance, an unusual distractor will be stronger to attract the attention than a usual one (Second Scenario). These observations are in line with the evidence indicating that forcing the subject to orient his attention to the target, before its presentation, can reduce the effect of novelty distraction in an audio-visual task (Parmentier et al., 2008).

The results are also consistent with the effect of IC in complex systems that have more than one basin of attraction. If the IC is exactly in one of these basins of attraction, the system usually stays in this basin (First Scenario). However, if the initial condition is in the middle of these basins, the one that has the steeper slope (higher level of attraction) will determine the behavior of the system (Second Scenario). Nevertheless, these deductions cannot be extended to other senses. For instance, the novel tactile distractions had no effect on the accuracy of the responses (i.e. correct inhibitory and excitatory response). However, it could increase the reaction time despite the pre-orientation of the attention to the target (Parmentier et al., 2011). It means that the effect of initial condition can be violated when there are visual targets and tactile distractions.

In another study on the effect of attention direction on the novelty processing, it was shown that processing of novel (unusual) events does not necessarily need attention (Schomaker & Meeter, 2014) that was consistent with the results of other studies indicating that direction of attention lacks strong effect on the processing of novelty (Chong et al., 2008; Tarbi, Sun, Holcomb, & Daffner, 2011). The results of these studies can also justify our observation with regard to no significant difference between the effect of unusual and usual distractors. That is, the processing of novel features that are embedded in unusual images does not necessarily capture the attention.

It seems that the necessity of attending to novel characteristics of unusual stimuli is determined through a prioritization process. This result supports the idea of “top–down modulation of novelty processing” (Chong et al., 2008). Based on this idea, the circumstance of presenting a novel (unusual) event determines the amount of attention that should be allocated to it. Therefore, it can be claimed that in the competition between novel features of unusual images and the predefined goal, the prioritization process gave the higher priority to the goal in our experiment. In other words, the results show that goal-directed attention puts the priority on the predefined position of goal appearance and ignores the appearance position of any kind of distraction (usual or unusual).

Therefore, we can claim that both kinds of distraction have approximately the same effect on goal-directed attention. However, there are several other possibilities about the mechanism of the human attention control system that can lead to the observation of no significant difference between the effect of usual and unusual distractors. Any kind of distraction can surprise participants, because these are unexpected. We also believe that unusual distractors have more surprise effect than usual ones, due to their novel features that have never been experienced by the participant. However, results show that increasing the amount of surprise does not necessarily lead to the same increment of attention allocation. Therefore, it is possible that in our attention control system, there is a logarithmic or bell-shaped relationship between the amount of surprise and the quantity of required attention. The other possibility is that usual images contain a significant amount of bottom-up surprise (Itti & Baldi, 2009) that dominates the amount of novelty embedded in unusual images.

This possibility may also exist that the surprising effect of the distraction and novel features of unusual images do not occur in parallel. It seems that distraction makes its effect first and novelty later. Since the novelty of unusual distractors is detected by delay, the response of the subject is just affected by the distraction. In other words, the contextual novelty of unusual images creates its effect later, i.e. after the subject’s response. Investigation of overt attention showed that the first fixations were on the position of target, and participants did not immediately change their fixation into the position of unusual distractors. On average, they leave the location of target appearance after about 1149 (SD=226) ms. It seems that overt attention to novel features of distractors has a considerable delay, but we cannot say anything about covert attention with certainty. Therefore, the next possibility is worth investigating.

Both distraction and novelty compete to make their surprising effect simultaneously. However, according to the Kahneman’s limited capacity model of attention (Kahneman, 1973), our central processor evaluates the demands made by distraction and novelty and adjusts the attention toward one of them (i.e. distraction demand). In other words, there are two objects that try to capture the subject’s attention in unusual distractors: distraction and novelty. Due to the limited capacity, our attention system selects one of the aspects (i.e. distraction) for processing and filters the other aspect (i.e. novelty) (Broadbent, 1987). Therefore, both kinds of distractors could have had the same effect.

All discussed possibilities show the circumstances that both usual and unusual distractors can have similar effects on the performance of goal-directed attention. The analysis of the power also demonstrated that to reveal the effect of the distractor type on the attention performance, a large sample is required. Therefore, it seems that the effect of distractors type on the goal-directed attention is extremely small.

Besides, the main purpose of the study was to show the attraction difference between usual and unusual distractors when the IC of attention was on targets’ location. Our results also showed that there was no difference between the performance of subjects in trials with distractors (usual or unusual) and without any distraction. The impairment effect of distraction in a goal-directed attention task was shown in different studies (Beaman et al., 2014; Rosenberg, Noonan, DeGutis, & Esterman, 2013). Therefore, it seems that our results are inconsistent with this proved impairment effect. Our experiments started with two trials with no distractor.

All participants responded to these two trials with 100% accuracy. Therefore, if we just consider these two trials, the impairment effect of distraction is revealed. However, there is still the question of why participants had a considerable error in the following trials without distractor, which is approximately equal to the error in trials with distractor. Two-thirds of the trials contained distractor (usual or unusual) and participants are likely to be biased towards trials with distractors. In other words, since most of the trials (2/3) have a distractor, participants may attributed lower probability to the occurrence of trials with no distractor.

When they observed a trial with no distractor, they might experience a kind of surprise that led to the incorrect response. We call this kind of surprise as “biasing surprise.” Our observations showed that in contrast with the subject expectation to observe a distraction in the next trial, they had no saccade to the top left of the screen (the position where distractors are presented) in trials with no distractors. Consequently, we can claim that biasing surprise involves with the subject’s covert attention. It is due a mismatch between the subject’s expectation (bias) and the input proposition.

Therefore, biasing surprise is nearly the same as “mismatch-based surprise” introduced by Lorini, and Castelfranchi (Lorini & Castelfranchi, 2006). It challenges the subject’s expectation about the probability of the occurrence of trials with no distractor. Therefore, the subject needs some mental efforts, which activates the prefrontal cortex (Cavanagh, Eisenberg, Guitart-Masip, Huys, & Frank, 2013) to overcome the power of bias and reconstruct the probabilities. This effort is called “counteracting bias” by Minamimoto et al. (Minamimoto, Hori, Yamanaka, & Kimura, 2014).

In addition to biasing surprise, there is another possible reason for observing the nearly equal effects between trials with distractor and with no distractor. It has been shown that when there is no common feature between target and distraction, the impairment effect of distraction on reaction time is not considerable (Treisman & Gelade, 1980). In our experiments, the predefined target had highly different shape, color, orientation, and contextual meaning from the distractions. There was no confusion between them. Therefore, there was a negligible effect on the efficiency of responses. Consequently, the participants’ performance was approximately similar in either presence or absence of distractors.

The results of this preliminary study demonstrated the importance of the initial focus of the attention. These outcomes can help teachers or the lectures to design their presentation to optimally guide the initial position of the audience attention. They can change the IC of the attention to have more robustness against the existence disturbance in the environments. The interesting observation of the effect of bias can also be useful in neuroeconomic studies that aim to attract the customer attention. The results show that changing the statistical distribution of different objects’ representation can affect the human attentional performance.

Through a goal-directed attention performance test, we investigated the effect of usual and unusual distractors when the initial position of the attention was on the place of the goal and far from the attraction basin of the distractions. Results showed no significant difference between the effect of usual and unusual distractors on total percentage of the correct responses and the reaction time. The outcomes suggested the possible sensitivity of the attention control system to the IC that seems worthwhile to be considered in design of environments that demand people’s attention. Results also showed that subject bias reorients the attention by any event that is against predilection.

Although the theoretical explanation of the IC sensitivity and the outcomes of the statistical analysis showed the very low effect size of the distractors’ type on attention performance in the condition of pre-locating the attention on the targets, investigations with more subjects were required to find significance results.
